# Clinical and histopathological features of follicular thyroid cancer in Chile

**DOI:** 10.20945/2359-3997000000580

**Published:** 2023-01-17

**Authors:** René Díaz, José Miguel Domínguez, Hernán Tala, Roberto Olmos, Pedro Pineda, Daniela Olivarí, Marcela Jiménez, Ximena Mimica, Alejandra Lanas, Gerson Ocares, Jorge Sapunar

**Affiliations:** 1 Centro Oncológico Fundación Arturo López Pérez Departamento de Medicina Interna Unidad de Endocrinología Santiago Chile Unidad de Endocrinología, Departamento de Medicina Interna, Centro Oncológico Fundación Arturo López Pérez, Santiago, Chile; 2 Pontificia Universidad Católica de Chile Facultad de Medicina Departamento de Endocrinología Santiago Chile Departamento de Endocrinología, CETREN-UC, Facultad de Medicina de la Pontificia Universidad Católica de Chile, Santiago, Chile; 3 Clínica Alemana de Santiago Departamento de Medicina Interna Unidad de Endocrinología Santiago Chile Unidad de Endocrinología, Departamento de Medicina Interna, Clínica Alemana de Santiago, Chile; 4 Universidad del Desarrollo Facultad de Medicina Santiago Chile Facultad de Medicina de la Universidad del Desarrollo, Santiago, Chile; 5 Hospital Clínico de la Universidad de Chile Departamento de Medicina Interna Unidad de Endocrinología Santiago Chile Unidad de Endocrinología, Departamento de Medicina Interna, Hospital Clínico de la Universidad de Chile, Santiago, Chile; 6 Hospital Guillermo Grant Benavente Departamento de Medicina Interna Unidad de Endocrinología Concepción Chile Unidad de Endocrinología, Departamento de Medicina Interna, Hospital Guillermo Grant Benavente, Concepción, Chile; 7 Facultad de Medicina de la Universidad de La Frontera Departamento de Medicina Interna Unidad de Endocrinología Temuco Chile Unidad de Endocrinología, Departamento de Medicina Interna, Facultad de Medicina de la Universidad de La Frontera, Temuco, Chile; 8 Centro Oncológico Fundación Arturo López Pérez Departamento de Cirugía Unidad de Cirugía de Cabeza y Cuello Santiago Chile Unidad de Cirugía de Cabeza y Cuello, Departamento de Cirugía, Centro Oncológico Fundación Arturo López Pérez, Santiago, Chile; 9 Centro Oncológico Fundación Arturo López Pérez Departamento de Investigaciones Oncológicas Unidad de Investigaciones Clínicas y Epidemiológicas Santiago Chile Unidad de Investigaciones Clínicas y Epidemiológicas, Departamento de Investigaciones Oncológicas, Centro Oncológico Fundación Arturo López Pérez, Santiago, Chile

**Keywords:** Thyroid cancer, follicular, diagnosis, prognosis

## Abstract

**Objective:**

Follicular thyroid carcinoma (FTC) is less frequent but has a worse prognosis than papillary carcinoma. The available evidence on pre-operative characteristics of FTC is controversial. Our objective was to characterize the clinical, ultrasound and histopathological presentation of FTC patients treated Chile.

**Subjects and methods:**

Retrospective analysis of 97 patients treated for FTC in 6 large centers in Chile. We analyzed their ultrasonographic features and classified the nodules according to ATA risk of malignancy and TI-RADS score, as well as the cytological findings according to the Bethesda system. We described their clinical and histopathological findings at diagnosis and classified their risk of recurrence and mortality according to ATA 2015 recurrence risk category and the eighth edition of the AJCC/UICC staging system, respectively.

**Results:**

Median age was 48 years and 73.2% were females. The median diameter was 38.8 mm; only 9.5% of them were microtumors. According to ATA risk of malignancy, 86% of the nodules were low or intermediate suspicious, while 78% were category 3 or 4A nodules according to the TI-RADS. Regarding the Bethesda system, 65.9% had indeterminate cytology (20.6% category III and 45.3% category IV). At histological examination, most were minimally-invasive and angio-invasive tumors with less than 4 foci (54.7% and 28.4% respectively). More than 90% of FTC were unifocal and there was no lymphovascular or extrathyroidal invasion or lymph node involvement. Four patients (4.1%) had distant metastases at diagnosis. Most patients (95%) had stage I or II disease according to the AJCC/UICC staging system, while the risk of recurrence was low at 51.5% when using the ATA risk of recurrence scale.

**Conclusions:**

At diagnosis, most FTCs were nodules of low or intermediate suspicion at ultrasound, nearly two thirds had indeterminate cytology according to the Bethesda system, and nearly 50% of them were of low risk of recurrence.

## INTRODUCTION

Thyroid nodules are a very common finding in the general population. Although the frequency of palpable nodules is around 5%, it can increase to more than 50% when screened with thyroid ultrasound, particularly in the elderly (1). Thyroid nodules are benign in about 90% of cases and most of them only need ultrasound follow-up. In patients with a family or personal history of risk to develop thyroid cancer and/or with nodules that meet some ultrasound characteristics, a cytological study by fine needle aspiration (FNA) should be performed to rule out cancer (2,3). The nodule size requiring FNA depends on its ultrasound characteristics and risk of malignancy. Generally, guidelines do not recommend aspiration for nodules < 1 cm, regardless of their ultrasound characteristics, except for suspicious adenopathies, suspected extrathyroid extension or some characteristics suggesting higher risk in the case of growth of a suspicious nodule.

The most common thyroid cancers are well-differentiated cancers from the follicular epithelium, including papillary thyroid carcinoma (PTC), follicular thyroid carcinoma (FTC), invasive encapsulated follicular variant papillary carcinoma and oncocytic carcinoma of the thyroid (OTC). They all share the ability to concentrate radioiodine but differ in epidemiology, clinical behavior, ultrasound appearance, cytopathological findings, and molecular alterations (2).

Clinical guidelines for the diagnosis and treatment of thyroid nodules and differentiated cancer have focused mainly on PTC due to its higher frequency (>90% of all thyroid cancers); on the other hand, the available evidence regarding FTC is controversial, mainly with regard to its preoperative diagnosis (2).

It is difficult to assess the epidemiological evolution of FTC due to changes in the criteria for the histopathological diagnosis of PTC. In the sixties a thyroid cancer with a >50% follicular structure was considered to be a FTC. Subsequently, it was established that nuclear alterations allowed PTC to be differentiated from FTC, so that the latter would be a malignant epithelial tumor with a follicular differentiation pattern but without nuclear characteristics of PTC; when these alterations were present it would be a follicular variant of PTC. Thus, as the recognition of nuclear alterations in PTC increased, the incidence of FTC decreased, with PTC currently being the most frequently diagnosed thyroid cancer (4–6). Chilean studies have found a similar situation, where 91.8%-96.3% of the thyroid cancers are PTC and only 2.4%-5.1% are FTC (7,8).

It has been suggested that iodine excess may be a weak promoter of thyroid carcinogenesis and that high iodine intake is associated with higher ratios of PTC: FTC (9). In Chile, salt iodination started in 1979 and initially required the addition of 100 µg of iodine per kilogram of salt. In 2000, after finding an excess of iodine in school-aged children, this addition was reduced to 40 µg of iodine per kilogram of salt (10). Currently, Chile is considered to have optimal intake of iodine in children and adults (11).

The new 5th edition of the World Health Organization (WHO) Classification of Endocrine and Neuroendocrine Tumors recognizes the existence of 3 variants of follicular thyroid cancer: minimally-invasive FTC (tumor capsular invasion only), angioinvasive FTC and widely invasive FTC and each type has a different prognosis (12). Unlike PTC, recurrence in FTC generally presents as distant metastases (mainly to the lungs and bones); cervical lymph node involvement is infrequent, which also confers a worse prognosis. The frequency of distant metastases in FTC varies from 3% to 30% in published clinical series, a range that reflects the heterogeneity of cases included (some series include as FTC cases of Hürthle-cell cancer or poorly differentiated cancer, which have a different clinical course). As is the case with PTC, the response to radioiodine treatment is good, but rarely curative in the presence of large distant metastases (5).

As for preoperative study, the most suggestive ultrasound findings of PTC show a nodule that is taller than wide on cross-sectional images, hypoechoic, solid, with irregular or poorly defined margins, and the presence of microcalcifications. However, these ultrasound characteristics are rare in FTCs, which usually appear on ultrasound as large nodules without microcalcifications. Furthermore, as previously noted, lymph node metastases in FTC are rare, even in widely invasive FTCs (2,3,13). Generally, ultrasound findings do not permit to distinguish a follicular adenoma (FA) from a FTC, although it has been suggested that the male gender, a larger size and hypoechoic nodules, without halo and without cystic changes have a higher risk of malignancy (14).

In nodules that are studied by FNA, cytology results must be reported according to the Bethesda system, which classifies cytological findings into six categories, with FTCs usually reported as Bethesda III or IV, which corresponds to indeterminate cytology. On the contrary, due to its nuclear alterations, PTC can be identified quite accurately through cytology. This occurs because malignancy in FTC is determined by evidence of invasion, either capsular or vascular, for which a complete histopathological analysis of the tumor is required, this being one of the main limitations of thyroid cytology to diagnose this type of disease. Cancer. The risk of malignancy for a thyroid nodule with a cytology reported as follicular neoplasia (Bethesda IV) ranges between 10% to 40% and from 6% to 28% if it has been reported as Bethesda III. Some authors have proposed that the presence of some elements in cytology such as hypercellularity, nuclear overlap, and nuclear atypia suggest a FTC rather than an adenomatous goiter or FA (15).

Our objective was to characterize the clinical, ultrasound and histopathological findings of a series of patients with FTC from 6 Chilean centers with similar management of the disease.

## MATERIALS AND METHODS

We collected a series of histologically-confirmed FTC cases, excluding OTC, operated since 2015 in 6 large head-and-neck-surgery centers in Santiago and other regions of Chile where descriptive information of preoperative ultrasound was available.

The data required for analysis were obtained from the records of pathology services, thyroid cancer registries and clinical records of the participating centers, with the approval of the Scientific Ethics Committee of the *Instituto Oncológico Fundación Arturo López Pérez*, which is nationally accredited and its resolution accepted by the Ethics Committees of the other participating centers. The aforementioned Ethics Committee decided to grant an informed consent waiver considering that most of the information was extracted from records and that the data of the clinical record were obtained by the treating physicians and tabulated by encrypting the identity of the patients.

We collected demographic variables (age, gender), characteristics of the suspicious nodule on pre-operative echotomography (largest diameter, shape, echogenicity, margins, vascularization pattern, presence and pattern of hyper-echogenic “spots”, presence of suspicious adenopathies), ultrasound risk of malignancy (TI-RADS and American Thyroid Association – ATA – scales) (2,16), classification of cytopathological findings at pre-operative needle biopsy (Bethesda classification) (17), type of surgery performed and histopathological findings (tumor size, multicentricity, degree of invasion, extrathyroid involvement), presence of distant metastases and staging according to the AJCC Cancer Staging Manual 8th edition (18). The risk of recurrence was estimated using the scales of the ATA and the “Clinical Protocol for the management of differentiated thyroid cancer 2020” of the Chilean Ministry of Health (MINSAL) (19).

Categorical variables are presented as number and percentage; continuous variables are presented as mean and standard deviation or as median and confidence interval, as appropriate. Categorical variables were compared using Fisher's exact test, and continuous variables were compared using Student's t-test or Mann-Whitney u-test as appropriate. A p value lower than 0.05 was considered significant. For data analysis, the STATA 14.0 program (v.16.0: College Station, TX: StataCorp LP) was used.

## RESULTS

Ninety-seven histopathologically-confirmed FTC cases were included, operated between 2015 and 2019 in head-and-neck-surgery centers in Santiago (n = 4) and Chilean regions (n = 2). The median age was 48 years (95% CI 41-52); 71 patients (73.2%) were women. There were no significant differences in the median age between genders (men 51 years (95% CI 41-63) vs. women 46.5 years of age (95% CI 41.3-51.6), p value = 0.3406).

Table 1 summarizes the main findings at pre-operative thyroid echotomography and details the number of cases for which the variable could be obtained. The median largest diameter of suspicious nodules was 36 mm, only 9.5% of them were micronodules (<1 cm in diameter). Most nodules were wider than tall, hypoechoic, with a regular margin, without hyperechoic spots, extrathyroid extension, or suspicious adenopathies. When applying the ATA scale, 86% of the nodules were suspicious for low or intermediate malignancy, while applying the TI-RADS scale 78% of them were category 3 or 4a nodules.

**Table 1:** Ultrasound characteristics of patients with follicular thyroid carcinoma operated between 2015 and 2019 in 6 head-and-neck-surgery centers in Chile

Echogenicity (n = 58)	%
Isoechogenic	24.1
Hyperechogenic	12.1
Hipoechogenic	63.8
**Margins (n = 66)**	**%**
Regular, complete	80.3
Regular, incomplete	4.5
Lobulated	6.1
Irregular	9.1
**Hyperechogenic spots (n = 64)**	**%**
No	68.6
Comet-tail artifact	6.3
Macrocalcification	9.4
Calcified halo	7.8
Microcalcifications	7.9
**Nodule size (n = 52)**	**%**
Wider	82.7
Longer	17.3
**Extra-thyroid extension (n = 68)**	**%**
No	95.6
Yes	4.4
**Suspicious adenopathies (n = 63)**	**%**
No	97.2
Yes	2.8

According to the Bethesda classification twenty (20.6%) were category III and 44 (45.3%) were category IV nodules. Six (6.2%) were category II and of these, five were category 3 on the TI-RADS scale. All patients with category II of Bethesda classification had very low or low recurrence risk in ATA scale.

Table 2 summarizes the main surgical findings. Eighty-six (90.5%) patients underwent total thyroidectomy, the tumor size was 30 mm (95% CI 23.1-32) and 86 (90.5%) of the tumors were ≥10 mm. There was a good correlation between the surgical finding and the size of the suspicious nodule on the pre-surgical echotomography (r = 0.6589, p value <0.0001). As for histology, most FTCs were minimally-invasive and angio-invasive tumors with fewer than 4 foci (54.7% and 28.4% respectively). Mitosis or necrosis were only described in less than 10% of the histopathological reports. In over 90% of cases, the lesion was unifocal and there was no lymphovascular or extrathyroid invasion. Only one (1.1%) patient has lymph node involvement and four (4.1%) patients had distant metastases at the time of diagnosis (3 to the lungs and 1 to the bones). We did not find association between ultrasonographic features and the presence of both lymph nodal and distant metastases.

**Table 2:** Post-surgical histological findings in patients with follicular carcinoma of the thyroid operated between 2015 and 2019 in 6 head-and-neck-surgery centers in Chile

Type of surgery (n = 95)	%
Lobectomy	6.3
TT	90.5
TT+ND	3.2
**Tumor diameter (n = 95)**	**Me (CI 95%)**
	30 (23.1- 32)
**Tumor size (n = 95)**	**%**
≤1cm	9.5
>1cm	90.5
**Histology (n = 95)**	**%**
Minimally invasive	54.7
Angioinvasive < 4 foci	28.4
Angioinvasive ≥ 4 foci	2.1
Widely invasive only capsule	11.6
Widely invasive angio + capsule	3.2
**Multicentric (n = 95)**	**%**
No	92.6
Yes	7.4
**Extra-thyroid extension (n = 92)**	**%**
No	95.6
Microscopic	2.2
Macroscopic	2.2
**Mitosis (n = 63)**	**%**
No	95.2
Yes	4.8
**Necrosis (n = 75)**	**%**
No	90.7
Yes	9.3
**Linfovascular Invasion (n = 90)**	**%**
No	96.6
Yes	3.4
**Surgical Margin (n = 92)**	**%**
No	94.6
Yes	5.4
**Nodal involvement (n = 95)**	**%**
No	98.9
Yes	1.1
**Distant metastases (n = 97)**	**%**
No	95.9
Lung	3.1
Bone	1.0

Me: median TT: total thyroidectomy; ND: lymph node dissection.

Table 3 shows the relative importance of staging categories according to the AJCC Cancer Staging Manual 8th edition and recurrence risk categories using the ATA and Chilean Ministry of Health's “Clinical Protocol for the management of differentiated thyroid cancer 2020” scales. About 95% of the patients had stage I or II disease; less than 5% of them had stage IVB disease, while the risk of recurrence was very low or low in 44.3% of cases when using the MINSAL 2020 scale and low in 51.5% when using the ATA scale.

**Table 3:** Recurrence risks in patients with follicular carcinoma of the thyroid operated between 2015 and 2019 in 6 head-and-neck surgery centers in Chile

AJCC VIII (n = 97)	%
I	85.5
II	10.3
IVB	4.2
**MINSAL risk scale (n = 97)**	**%**
Very low	20.6
Low	23.7
Intermediate	46.3
High	9.4
**ATA risk scale (n = 97)**	**%**
Low	51.5
Intermediate	39.1
High	9.4

MINSAL: Chilean Ministry of Health.

## DISCUSSION

This is the first national study to describe FTC presentation. As reported in literature, epidemiological characteristics show the predominance of female patients and diagnosis in the fifth decade of life, at a slightly older age than PTC (20).

As for ultrasound findings, over 90% of FTCs were larger than 1 cm and over 85% had low or intermediate ultrasound risk according to the ATA and TI-RADS scales (2,16). This finding is consistent with previously described experiences, and occurs because most FTCs are single, intrathyroidal nodules, generally iso or hypoechoic, with regular margins, without internal calcifications or cervical lymphadenopathy. This could explain why most FTCs in our series, as well as those previously reported, are >1 cm in size, since the indication for FNA in these cases is probably determined by the size of the nodule and not by the presence of suspicious characteristics (7,20). This represents an additional clinical challenge because, at present, when the indication to FNA is defined according to the ultrasound characteristics of the thyroid nodules, there is a potential risk of underdiagnosing FTCs. However, this does not mean that nodule FNA is required more than indicated by guidelines, since studies show that small FTCs have a very low risk of recurrence, angioinvasion and mortality.

In our series, 81% of FTCs were diagnosed as FNA Bethesda III or IV. As to cytology, FTC cannot be differentiated from follicular adenoma, since complete resection of the lesion and its histological study are required to define the presence of signs of malignancy such as angioinvasion or capsular invasion (20). Follicular neoplasia, which consists of a combination of high cellularity, with microfollicular architecture and little or no colloid, is a cytological term that encompasses both benign and malignant proliferation of follicular cells (21). In this sense, it is expected that in our series, as well as in previously published experiences, most patients have Bethesda-IV cytology (22). Additionally, considering the variability in the application of the Bethesda scale and interobserver variability, with a significant overlap between categories III and IV, it is not surprising that category III is the second most frequent one (23).

In our series 9% of FTCs were Bethesda-II FNAs, highlighting the difficulty in diagnosing FTC and the need to integrate clinical, radiological and cytological information to define the most adequate management for each patient (24).

The histological classification of FTC is controversial. The latest version (2022) of the WHO for the classification of tumors to endocrine organs classifies FTCs into three groups: minimally invasive when there is only capsular involvement; angioinvasive FTC in cases with venous vascular invasion (with or without capsular involvement) and widely invasive FTC in cases with evidence of extensive capsular involvement, usually with macroscopic extrathyroid extension, independent of vascular involvement. However, there is still controversy about the definition of angioinvasion and the great limitation of this classification is that it does not quantify the degree of vascular involvement, which is one of the main prognostic factors for distant metastasis and mortality in FTC (2,12,25). In order to highlight the prognostic role of angio-invasion, the 2015 classification of Armed Forces Institute of Pathology (AFIP) divides FTC with vascular invasion into those with limited angio-invasion (<4 foci) and those with extensive angio-invasion (≥4 foci) (26). In our series, almost 17% of FTCs presented extensive capsular and/or vascular involvement, which gives them a higher probability of developing distant metastases during follow-up.

As previously described, we found a low frequency (1.1%) of cervical lymph node involvement at diagnosis, which is a characteristic of FTC presentation. However, our patients had a lower percentage of distant metastases: 4.5% vs. 10%-15%. This finding may be due to the fact that our series comprises a majority of minimally invasive FTCs (54.7%) or FTCs with <4 foci of angio-invasion (28.4%), both conditions associated with a lower probability of distant metastasis (27). The low frequency of distant metastases and loco-regional extension, as well as a median age at diagnosis of 48 years in our series, explain that 91% of the patients were stage I or II according to the 8th edition of the TNM/AJCC classification (18).

Regarding the extent of surgery, 90% of patients underwent total thyroidectomy and only 3% underwent lymph node dissection, which is consistent with the low percentage of cervical lymph node involvement previously described. Only 6% of patients were managed with lobectomy, a figure that should increase considering that 81% of FTCs in our series had indeterminate FNA (Bethesda III or IV), where the management consensus indicates partial surgery if the preoperative evaluation allows it (24). On average, 15%-25% of the nodules with indeterminate cytology that are operated on are malignant. About 60% of them are PTC, of which up to 10% require totalization (28–30). Between 10%-15% are FTC; information on indication to totalization is scarce in this setting (27,28). In our series, of the 64 nodules with indeterminate cytology, 56 (87.5%) had a low risk of recurrence (minimally invasive FTC or angio-invasion <4 foci), so they did not require radioiodine ablation and, consequently, can be managed with lobectomy (18).

One of the limitations of this study is that, given its retrospective nature, we do not have information regarding the administration of radioiodine. There is little information and much controversy regarding the benefit of RAI in the low-risk FTC group, so its use should be selective, considering variables such as post-operative non-stimulated thyroglobulin, imaging studies (cervical ultrasound and others defined in each case) and patient preference (18). For example, RAI has not shown any benefit in terms of recurrence in minimally invasive FTC (24,31). In terms of recurrence risk, both on the ATA and MINSAL scales, about 50% of the patients had low or very low recurrence risk FTCs, while 40%-45% had intermediate risk and 9% had high risk disease, which could reduce the indication for radioiodine to about half of the patients included.

In conclusion, we present the first multicenter series that shows the clinical, ultrasound and histopathological characteristics of FTC in Chile. We emphasize that most of them were nodules of low or intermediate suspicion on ultrasound, more than 80% of them diagnosed in nodules of indeterminate cytology, and around 50% of FTCs had a very low or low risk of recurrence.
